# A preliminary account of *Culicoides* (Diptera: Ceratopogonidae) from the Andaman and Nicobar Islands with 13 new records and updated species inventory from India

**DOI:** 10.1186/s13071-024-06614-w

**Published:** 2025-01-17

**Authors:** Koustav Mukherjee, Surajit Kar, Atanu Naskar, Chandrakasan Sivaperuman, Dhriti Banerjee

**Affiliations:** 1 Diptera Section, Zoological Survey of India, Kolkata, West Bengal India; 2 Andaman- and Nicobar Regional Centre, Zoological Survey of India, Haddo, Port Blair, 744102 India

**Keywords:** Bluetongue virus, *Culicoides*, New records, Checklist, Andaman and Nicobar Island, India

## Abstract

**Background:**

The detection of multiple bluetongue virus serotypes, increasing trend in livestock density, rich biological diversity with high endemism, and the status of the Andaman and Nicobar Islands as a popular tourist destination underscore the need for a faunistic survey of medically and veterinary significant vector species, specifically *Culicoides*, in this region. Moreover, scattered information on Indian *Culicoides* species complicates the planning and implementation of preventive measures for pathogens transmitted by these vectors. This study aims to provide the first comprehensive account of the *Culicoides* fauna in the Andaman and Nicobar Islands, India, along with an updated checklist of Indian *Culicoides* species and their state-wise distribution.

**Methods:**

Surveys were conducted across various habitats in the Andaman and Nicobar Islands in September 2022 and 2023. Midges were collected using CDC light traps, light sheets, Malaise traps, and manual collection from exposed body parts of the author and volunteers (biting collection). Identification was carried out using relevant taxonomic keys and original descriptions. Additionally, an updated checklist of Indian *Culicoides*, based on published and grey literature from 1910 to the present, is provided, with an emphasis on their potential role in pathogen transmission.

**Results:**

A total of 3529 adult *Culicoides* were trapped during the survey, representing 5 subgenera and 3 unplaced species groups. The study recorded 23 *Culicoides* species, including 13 new species records for India: *C. barnetti*, *C. gouldi*, *C. flaviscutellaris*, *C. flavipunctatus*, *C. hui*, *C. histrio*, *C. guttifer*, *C. perornatus*, *C. okinawensis*, *C. quatei*, *C. obscurus*, *C. coronalis*, and *C. kusaiensis*.

**Conclusions:**

The Indian *Culicoides* fauna now includes 93 valid species, with many of them recognized as confirmed or potential vectors of important pathogens of animal health. The enriched species composition highlights the importance of systematic surveys in this island ecosystem and the need to determine the role of midges, if any, in pathogen transmission.

**Graphical Abstract:**

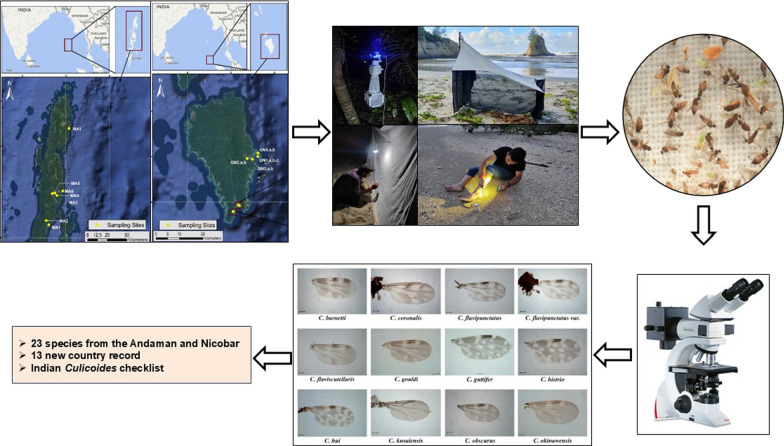

**Supplementary Information:**

The online version contains supplementary material available at 10.1186/s13071-024-06614-w.

## Background

*Culicoides* Latreille, 1809, a large genus of the family Ceratopogonidae with cosmopolitan distribution excluding New Zealand and Antarctica, includes species that are among the smallest hematophagous members of the order Diptera [1]. *Culicoides* biting midges are gaining significant attention due to their role as vectors of almost 60 viruses, 40 protozoans, and 24 filarial nematodes, impacting not only livestock and wildlife, but also humans [2, 3]. Furthermore, in some regions, many species within this genus are nuisance pests of humans and livestock, affecting forestry, construction, tourism, and other outdoor activities [1]. This hyper-diverse genus currently comprises 1347 valid species, of which 336 species are included in 38 species groups, 875 species are placed in 33 subgenera, and the remaining 136 species are not placed in any subgenus or group [4]. Despite being such a significant group in terms of holistic impact, the study on taxonomy, bio-ecology, and their role as vectors remain understudied when compared with other major dipteran vectors [1]. Investigation of the *Culicoides* fauna has been uneven across different regions of the world, with research on the European fauna probably being in a better state compared with research on other continents, where a large number of species are awaiting discovery [4]. In the Oriental realm, consolidated taxonomic data on *Culicoides* has never been compiled; however, a lot of information is available, usually on a country-wide basis [5–17]. One exception is Wirth and Hubert’s [18] monograph “The *Culicoides* of South-East Asia (Diptera: Ceratopogonidae)” which covered 168 species from multiple nations, including 53 new species, 8 subgeneric divisions along with effective taxonomic keys. However, a lot of work alongside systematic surveys remains to be done even in this region, as evident by constant discovery of new species and new country records [19, 20].

Taxonomic study of Indian *Culicoides* fauna began with Kieffer [21–24], followed by various authors such as Patton [25], Edwards [26], Smith [27], Mukerji [28], Smith and Swaminath [29], and Macfie [30]. After a long pause, a new generation of authors (Das Gupta & Ghosh [31], Sen & Das Gupta [6], Das Gupta [32–34], Choudhuri et al. [35], Majumdar et al. [36], Gangopadhyay & Das Gupta [37], Nandi et al. [38], Nandi & Mazumdar [39–41], Nandi [42], Nandi et al. [43]) made significant contributions to Indian *Culicoides* taxonomy. Prasad et al. [44] produced a comprehensive list of 40 *Culicoides* species and their state-wise distribution from India, which has since been updated to 73 species [42, 45, 46]. However, information on Indian *Culicoides* fauna appears to be livestock-centric and does not include various habitats and offshore islands.

The Andaman and Nicobar Islands are a chain of about 572 tropical islands situated in the west basin of the Andaman Sea, ranging between 6° and 14° north and 92° and 94° east [47]. The archipelago is a remarkable storehouse of biodiversity, sandwiched between two major biodiversity hotspots (the Indian subcontinent and the Malaysian–Indonesian region) [48]. As a result of being an isolated ecosystem, the endemism in flora and fauna is quite high [48]. Furthermore, the human inhabitants of the islands are a heterogeneous group that includes indigenous tribes (Jarawa, Onge, Great Andamanese and Sentinels of Andaman group of islands; Nicobarese and Shompen of the Nicobar group of islands), settlers from mainland India, and immigrants from nearby nations [49]. The archipelago, like many others in the Indian Ocean, has a complex geological and tectonic history that has shaped its formation over millions of years and is part of a larger geological feature associated with the convergent plate boundaries between the Indian and west Burmese plates (https://www.ndrdgh.gov.in/NDR/?page_id=790).

Recent increases in human settlement in the islands, and corresponding livestock populations, modernization of animal husbandry practices, intensification of construction practices, and an increased annual influx of tourists puts the island ecosystem at risk of various disease outbreaks ([50], https://www.and.nic.in/andaman/population.php; https://www.andamantourism.gov.in/tourist_data.php; 19th Livestock Census-2012). The island’s geographic isolation serves as a natural barrier to many diseases [51]. However, livestock-related incidents have occurred recently according to Sundar [52], livestock and poultry have been infected with a variety of parasite diseases after the 2004 tsunami. Additionally, *Culicoides*-borne diseases such as bluetongue virus (BTV) have been reported on these islands at a higher rate of seropositivity than in other states of India [51, 53]. Despite the above-mentioned facts, there has been no initiative to explore this island system for insect vectors of BTV.

This study provides a preliminary account of *Culicoides* diversity in the Andaman and Nicobar Islands and is the first study of the genus from the archipelago. This study also reviews previously published articles and provides a comprehensive species checklist of Indian *Culicoides* species with their geographic distribution and known pest status.

## Methods

### Study sites and collection procedure

**Fig. 1:**
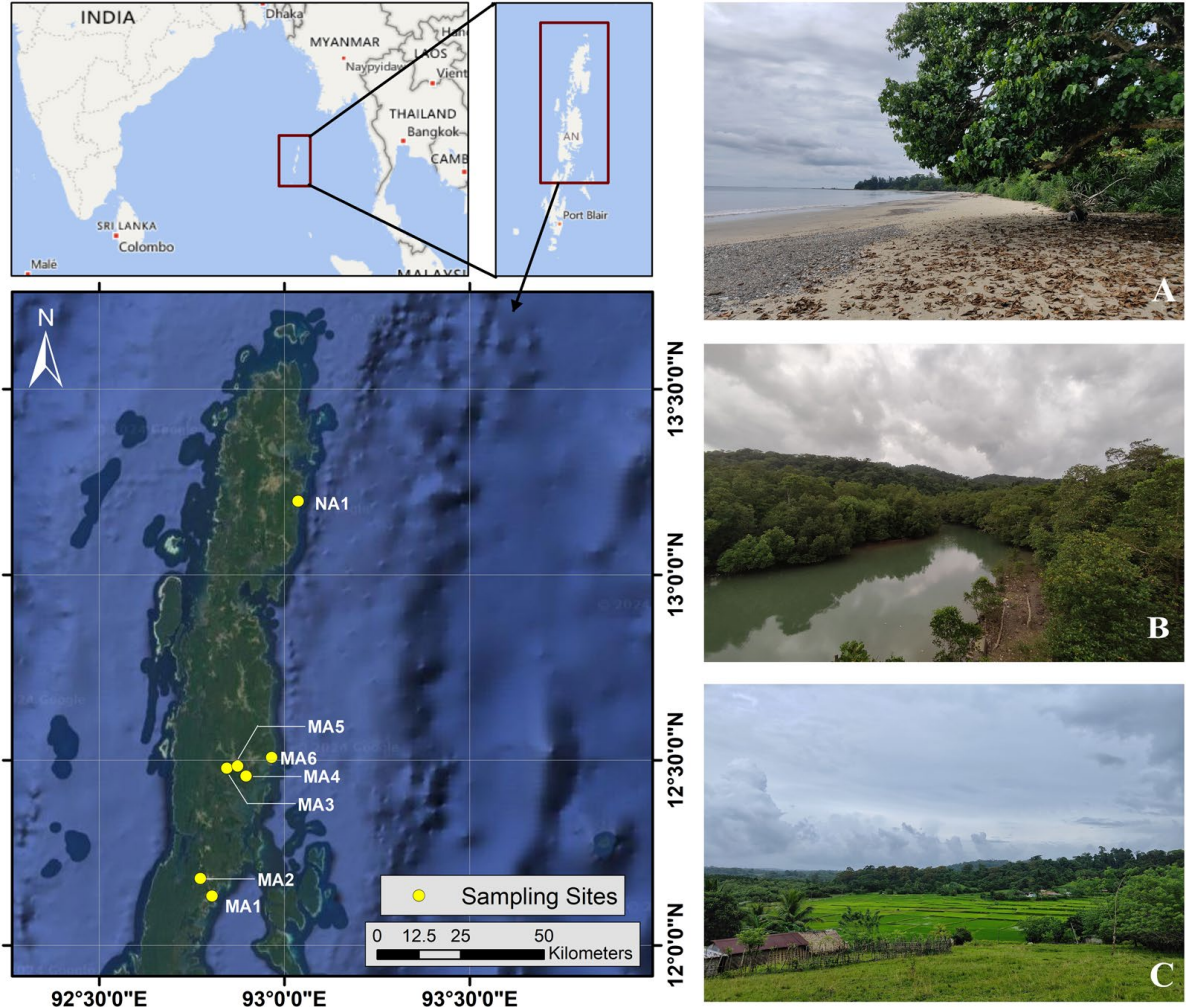
Map of sampling sites in North Andaman (NA1) and Middle Andaman (MA1-MA6). Representative photographs of some habitat type in the sampling sites: **A** White sandy beach with vegetation (MA6), **B** Mangrove (MA3), **C** Agricultural land with human settlement (MA5)

**Fig. 2:**
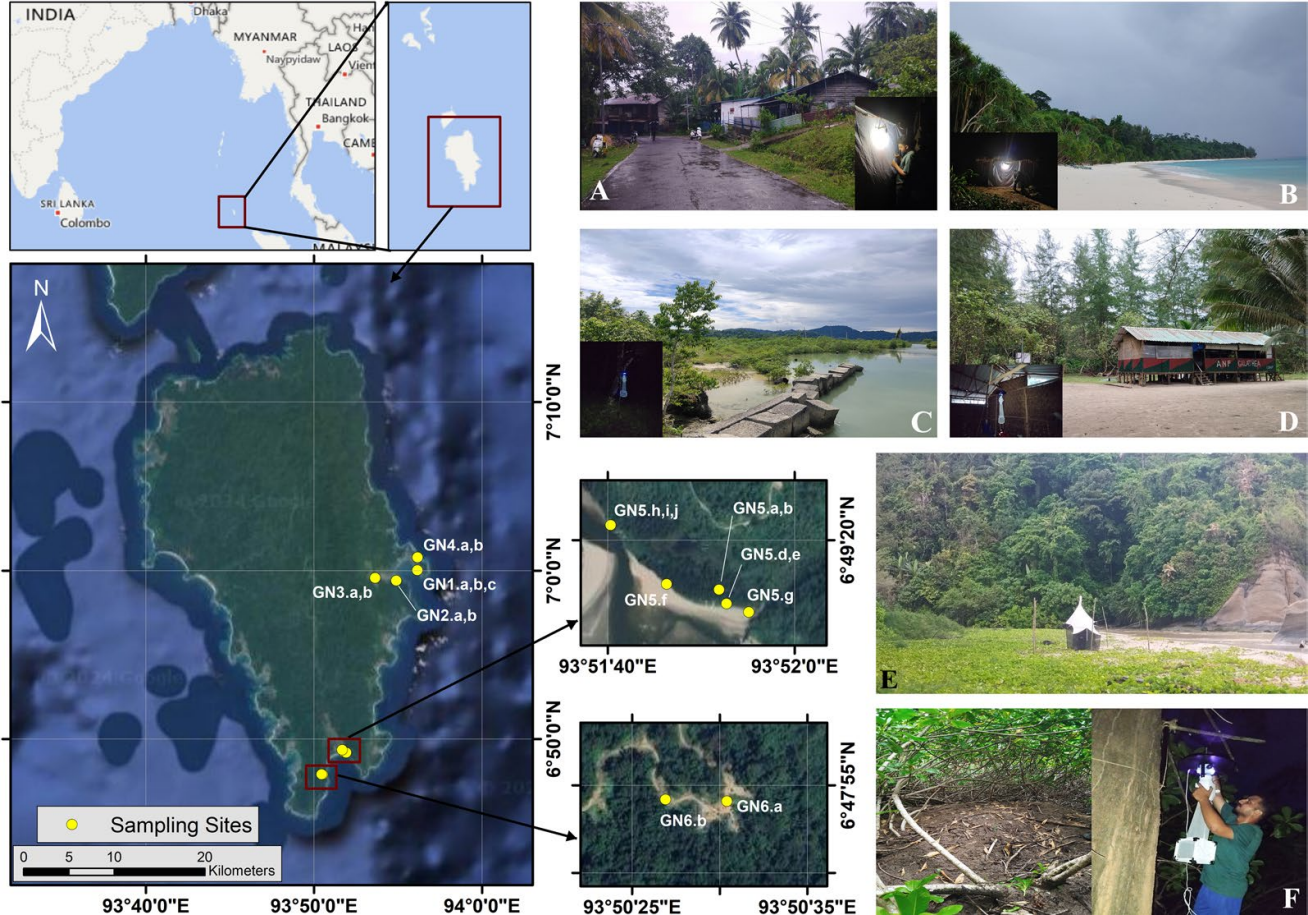
Map of sampling sites in Great Nicobar (GN1- GN6). Representative photographs of some habitat type in the sampling sites: **A** Area with human settlement (GN1.a- GN1.c, inset: LS), **B** White sandy beach with *Pandanus* shrubs (GN4.a- GN4.b, inset: LS), **C** Mangrove (GN3.a–GN3.b, inset: CDC), **D** Hut with residents (GN5.a–GN5.b,inset: CDC), **E** Malaise trap near an estuary (GN5.i–GN5.j), **F** Nicobar megapode(*Megapodius nicobariensis* Blyth, 1846) mound (GN5.f, inset: CDC)

In September 2022, an entomological survey was conducted in seven different sites of North Andaman (NA1) and Middle Andaman (MA1-MA6) Islands (Fig. 1, Table 1). The survey employed basic trapping methods such as sweep netting during day time and light collection at night. Using a white fabric sheet and a 12 W white LED light powered by a generator, the light sheet (LS) was operated for 4–5 h, usually between 5:30 p.m. and 10:00 p.m. *Culicoides* were collected from the sheet using a brush dampened with ethyl alcohol and stored in 70% ethanol. Furthermore, any midges biting the authors and volunteers present at the site of collection were also collected (Biting Collection: BC) using the same procedure. A second survey was conducted in September 2023 in six sites of Great Nicobar Island (GN1-GN6) (Fig. 2, Table 1). During this survey, a CDC light trap equipped with UV light (model-L1-MR-31a) was set roughly between 5:30 p.m. and 07:00 a.m. at a height of 1.7–2.2 m above ground along with a LS, which was placed at least 100 m away from the location of the CDC. A Malaise trap (MT) was also employed in a few sampling sites.

**Table 1:** Details of collection sites, trapping duration, and collection methods used to sample populations of *Culicoides* on the Andaman and Nicabar Islands

Collection site	Latitude	Longitude	Date of collection	Habitat type	Elevation	Site number	Collection method	Duration
MIDDLE ANDAMAN								
Baludera beach	12°08′00.7"N	92°48′15.2"E	17.09.2022	Collection site sandwiched between forest and Andaman Sea; no human or livestock in vicinity; two creeks on either side of trapping site (200 m approx)	1 m	MA1	LS, BC	05:31 p.m.–09:45 p.m.
Kanchangarh Bamboonullah	12°10′51.7"N	92°46′22.0"E	18.09.2022	Collection site adjacent to a mangrove creek surrounded by agricultural patches; a few human settlements with livestock present	2 m	MA2	LS, BC	06:03 p.m.–10:05 p.m.
Shyamkund Jetty, Bakultala	12°28′42.8"N	92°50′40.6"E	19.09.2022	Mangrove forest adjacent to a river with no human (except 2–3 fishermen at the time of collection) or livestock	4 m	MA3	LS, BC	05:30 p.m.–10:00 p.m.
Yeratta Mangrove plantation	12°27′25.5"N	92°53′45.9"E	20.09.2022	Area predominated by mangrove plantation and a barren area that gets flooded during high tide	0 m	MA4	LS, BC	06:15 p.m.–10:30 p.m.
Near Bharatpur forest	12°29′02.6"N	92°52′23.7"E	21.09.2022	Agricultural patch (paddy) between a small canal and forest; human and livestock present	3 m	MA5	LS	05:30 p.m.–10:00 p.m.
Aamkunj beach	12°30′27.7"N	92°57′54.8"E	22.09.2022	Collection site sandwiched between coconut plantation and Andaman Sea, acting as a tourist spot during day	0 m	MA6	LS, BC	05:15 p.m.–10:00 p.m.
NORTH ANDAMAN								
Saddle Peak National Park	13°11′52.1"N	93°02′11.6"E	25.09.2022	Heavily forested area (tropical evergreen forest)	2 m	NA1	LS	06:35 p.m.–09:45 p.m.
GREAT NICOBAR								
ALHW colony	7°00′02.3"N	93°56′09.2"E	15.09.2023	Area with human settlement	3 m	GN1.a	CDC	05:30 p.m.–06:30 a.m.
			17.09.2023	Area with human settlement		GN1.b	CDC	05:30 p.m.–06:30 a.m.
			17.09.2023	Area with human settlement		GN1.c	LS	06:30 p.m.–10:00 p.m.
Central Nursery, Magar nallah	6°59′25.2"N	93°54′53.6"E	16.09.2023	Heavily forested area (tropical evergreen forest)	42 m	GN2.a	LS	05:00 p.m.–10:00 p.m.
						GN2.b	CDC	05:30 p.m.–06:30 a.m.
Govind Nagar mangrove	6°59′34.9"N	93°53′38.0"E	18.09.2023	Mangrove area with tidal marshes and agricultural patches	11 m	GN3.a	LS, BC	06:30 p.m.–10:00 p.m.
						GN3.b	CDC	07:00 p.m.–06:30 a.m.
B-Quarry beach	7°00′48.0"N	93°56′09.9"E	19.09.2023	White sand beach with dense *Pandanus* shrubs; 140 m from nearest human settlement	0 m	GN4.a	LS	06:30 p.m.–10:00 p.m.
						GN4.b	CDC	06:40 p.m.–06:30 a.m.
Forest protection camp, Galathea wildlife range	6°49′14.7"N	93°51′51.9"E	21.09.2023	Inside a hut with three local residents; fowl, dogs and cats present	7 m	GN5.a	CDC	10:00 p.m.–07:00 a.m.
			27.09.2023			GN5.b	CDC	10:00 p.m.–07:00 a.m.
	6°49′13.2"N	93°51′52.7"E	21.09.2023	Mangrove area with swamp	5 m	GN5.d	LS	06:30 p.m.–10:00 p.m.
			22.09.2023			GN5.e	LS	06:30 p.m.–10:00 p.m.
	6°49′15.3"N	93°51′46.3"E	22.09.2023	*Pandanus* shrub near a Nicobar Megapode mound	0 m	GN5.f	CDC	07:00 p.m.–03:00 a.m.
	6°49′12.3"N	93°51′55.1"E	24.09.2023	White sandy region with a nearby small estuarine region	1 m	GN5.g	LS	06:00 p.m.–10:00 p.m.
	6°49′21.6"N	93°51′40.3"E	25.09.2023	Galathea river estuary	3 m	GN5.h	CDC	06:00 p.m.–05:00 a.m.
			21.09.2023–23.09.2023	Outside hut		GN5.i	MT	
			24.09.2023–27.09.2023	White sand beach with dense *Pandanus* shrubs		GN5.j	MT	
BRO 52 camp	6°47′54.1"N	93°50′30.4"E	23.09.2023	Cliff edge in BRO camp facing sea over a forest canopy	130 m	GN6.a	LT	06:00 p.m.–10:00 p.m.
	6°47′54.2"N	93°50′26.9"E	23.09.2023	Beside BRO quarter adjacent to a dense forest	125 m	GN6.b	CDC	06:00 p.m.–10:00 p.m.

*CDC* CDC-UV light trap, *LS* light sheet, *MT* malaise trap, *BC* biting collection, *BRO* Border Roads Organisation

During both surveys specimens were collected from a variety of habitat types including mangrove forests, sandy beaches, areas with dense human settlements and around isolated villages, agricultural patches, and in dense tropical evergreen forests, to ensure systematic coverage and to gain preliminary knowledge on *Culicoides* diversity in the islands (Table 1). However, due to erratic weather patterns and sudden torrential rainfall in the islands (average annual rainfall ≈ 3000 mm; average annual maximum and minimum temperature 32 °C and 22.1 °C, respectively; humidity levels 79–89%), the aforementioned collection procedures had to be tweaked according to field situations.

### Sorting, identification, and checklist preparation

*Culicoides* were initially separated from other insects under a dissecting microscope (Magnus MS 24; Leica EZ4). Midges were further segregated into morphospecies on the basis of wing pattern and thoracic coloration. For morphospecies found in at least two sampling sites, one female individual from each site was used for morphological identification, and for species collected from a single sampling site, at least two female specimens were used. Specimens for identification were mounted onto glass slides [54]. The head of each specimen was cleared in 10% KOH solution to improve visibility of features, such as sensilla coeloconica (SCo) and mandibular teeth. Slide-mounted specimens were measured [wing length, coastal length, palpal ratio (PR), eye separation, proboscis/head ratio (P/H), number of mandibular teeth, antennal ratio (AR), number of spines in the hind tibial comb, presence/absence and arrangement of sensilla coeloconica on an antennal segment, number and size of spermathecae] and observation of qualitative taxonomic characters (color of mesonotum and halteres, banding pattern of legs, wing pattern) under a compound microscope (Leica DM 1000). Wings were photographed using a camera (Leica K3C) attached to DM1000. Individual specimens were identified to species level using appropriate keys and illustrations [5, 13, 18, 42, 55, 56]. Some identified specimens were also matched with photographs of type and non-type specimens requested from museums and personal databases of authors. Some uncommon unidentified individuals were stored for future molecular studies and therefore are not a part of this report. No DNA-based identification approach was undertaken as all species reported here were readily identified using morphology. Problems arose with species belonging to subgenus *Trithecoides* as identification aids rely heavily on characters only visible on slide mount specimens, so there is a possibility of more species being present from this particular subgenus in our collection.

To prepare an updated species checklist, previously published articles and thesis relating to Indian *Culicoides* were collated using Google Scholar and PubMed^®^ and reviewed thoroughly in light of current taxonomic understanding and knowledge.

## Results

**Fig. 3:**
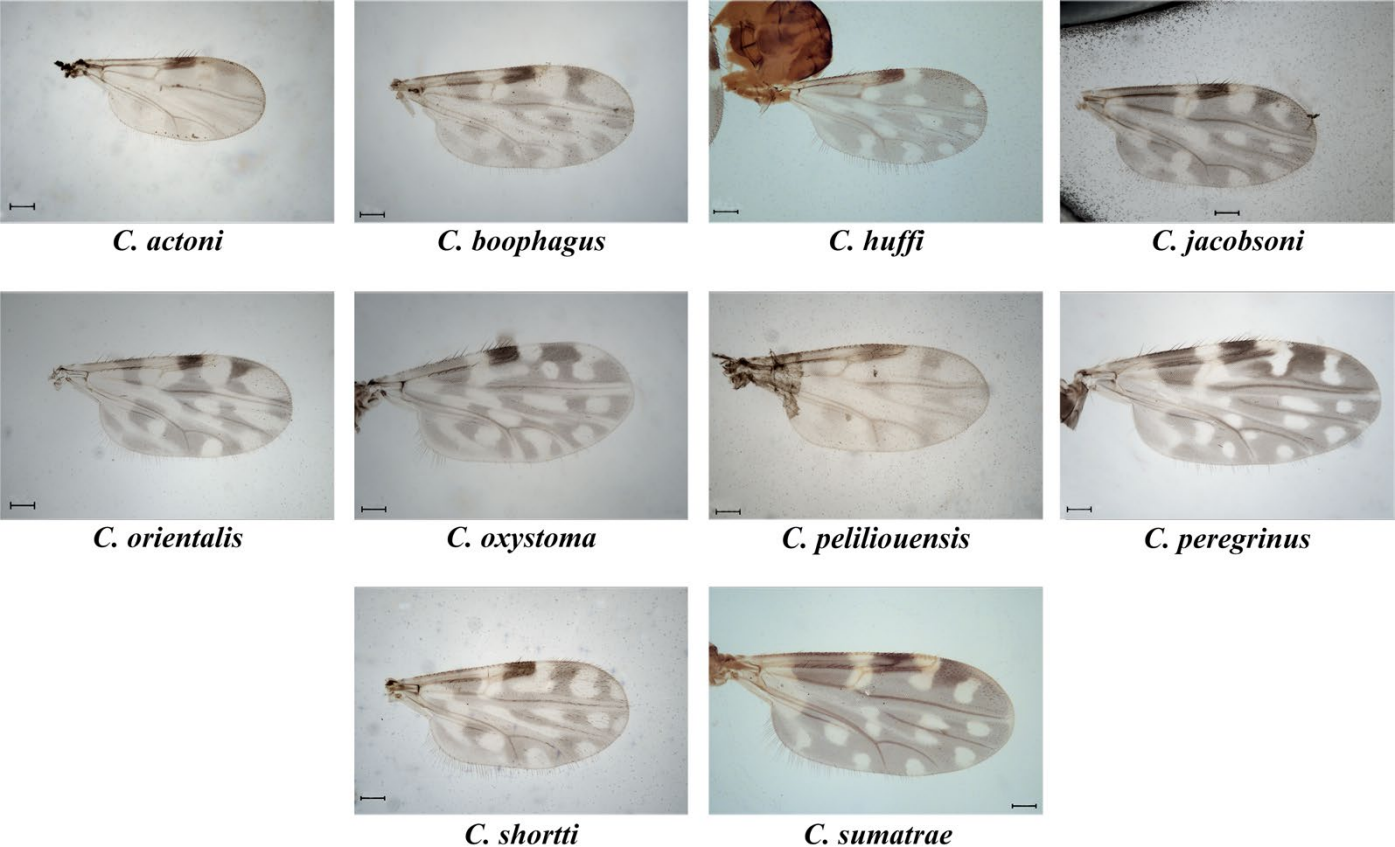
Wing photo plate of *Culicoides* species from the A&N, present in mainland India (scale bar 100 µm)

**Fig. 4:**
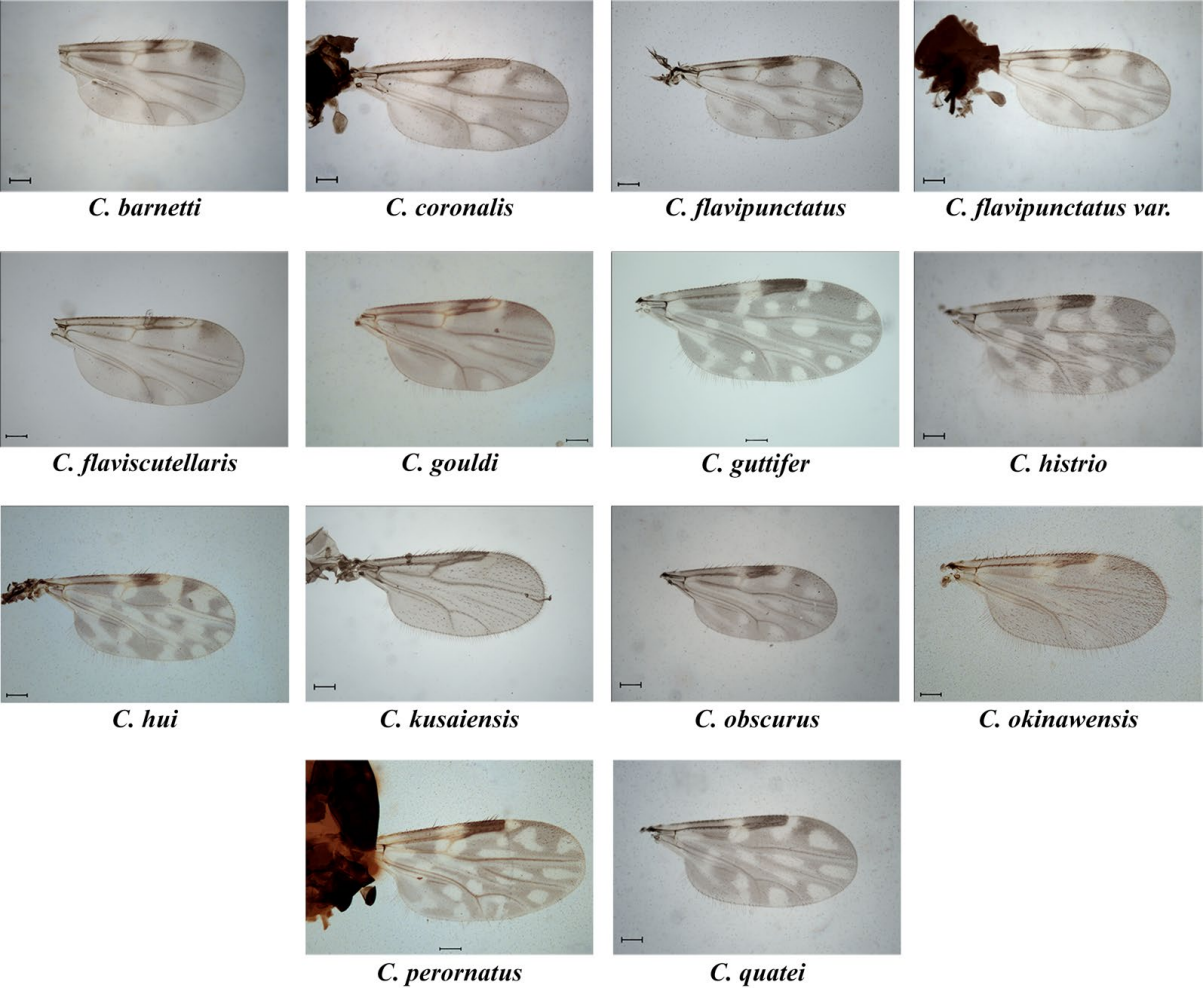
Wing photo plate of *Culicoides* species from the A&N, recorded first time from the country (scale bar 100 µm)

A total of 3529 adult individual *Culicoides* midges belonging to at least 23 species within 5 subgenera (*Hoffmania* Fox 1948, *Remmia* Glukhova 1977, *Avaritia* Fox 1955, *Trithecoides* Wirth and Hubert, 1959, *Meijerehelea* Wirth and Hubert, 1961) and 4 unplaced species groups (Clavipalpis Wirth & Hubert 1989, Ornatus Wirth & Hubert 1989, Coronalis Dyce et al. 2007, Kusaiensis Group Dyce et al. 2007) were collected during the study. Details of species collected using different methods viz., LS, CDC, BC, and MT, are provided in Tables 1 and 2. High rainfall and thunderstorms during the survey hampered sweep netting, and most of the specimens collected were damaged, thus we did not analyze the specimens collected using sweep net. In addition, several specimens with different morphotypes were also caught during this study, which were low in number and not identified to the species level as it was impractical to comment on taxonomic status without an integrative taxonomic approach. Further, accurate identification of the morphologically similar species and species belonging to subgenus *Trithecoides* requires more detailed analysis, and the results will be analyzed in future publications. Moreover, data regarding relative abundance, seasonality, and other ecological interactions will also be analyzed and published elsewhere. Among 23 species reported, 10 were recorded previously from mainland India, i.e., *Culicoides* (*Hoffmania*) *peregrinus* Kieffer, *Culicoides* (*Hoffmania*) *sumatrae* Macfie, *Culicoides* (*Remmia*) *oxystoma* Kieffer, *Culicoides* (*Avaritia*) *actoni* Smith, *Culicoides* (*Avaritia*) *jacobsoni* Macfie, *Culicoides* (*Avaritia*) *boophagus* Macfie, *Culicoides* (Avaritia) *orientalis* Macfie, *Culicoides shortti* Smith and Swaminath belonging to the Shortti group, *Culicoides huffi* Causey, belonging to the Clavipalpis group, and *Culicoides peliliouensis* Tokunaga, belonging to the Ornatus group (Fig. 3). The remaining 13 species were recorded for the first time from India (Fig. 4). Diagnostic characters of newly recorded species, their world distribution and remarks (if any) are presented below. Details of the species with their collection sites, reported pest status (Table 2), and material examined with morphometric measurements (Supplementary Table 1) are provided and all examined specimens are deposited in the National Zoological Collection (NZC) of Zoological Survey of India.

**Table 2:** *Culicoides* species collected during surveys of the Andaman and Nicobar Islands

Species	Site number	Pest status
*C. actoni* Smith, 1929	GN1.b, GN1.c, GN5.a, GN5.b, GN5.e, GN6.a	Proven vector of Bluetongue virus [2]; potential vector of filarial nematode *Onchocerca gibsoni* [18]; human biting [18, 65]
*C. barnetti* Wirth & Hubert, 1959	GN1.c, GN2.a, GN2.b, GN3.b, GN5.a, GN5.b, GN5.e, GN5.f, GN5.g, GN5.h, GN6.a	Human biting [18]
*C. boophagus* Macfie, 1937	GN5.a, GN5.b, GN5.e, GN5.f, GN5.h	–
*C. coronalis* Lee & Reye, 1955	MA1	Human biting [63]
*C. flavipunctatus* Kitaoka 1975	GN5.e, GN5.f, GN5.a, GN5.b, GN5.h, GN6.b	–
*C. flaviscutellaris* Wirth & Hubert, 1989	GN1.c, GN5.a, GN5.b	–
*C. gouldi* Wirth & Hubert, 1989	GN2.b, GN5.a, GN5.b, GN5.e, GN5.h	Human biting [18]
*C. guttifer* (de Meijere, 1907)	GN5.b, NA1	Potential vector of protozoan *Leucocytozoon caulleryi* in poultry [18]; potential vector of *Leishmania* [75]; human biting [18]
*C. histrio* Johannsen, 1946	GN3.a, GN3.b, GN5.a, GN5.b, GN5.e, GN5.f, GN5.g, GN5.h	Potential vector of *Thimiri orthobunyavirus* in birds [2] human biting [18]
*C. huffi* Causey 1938	GN1.a, GN1.b, GN1.c, GN2.b, GN3.b, GN5.d, GN5.e, GN5.h, NA1, GN6.a	Potential vector of avian *Trypanosoma* [76]; potential vector of *Leishmania* [67]; human biting [18, 65]
*C. hui* Wirth & Hubert 1961	GN5.a, GN5.b	–
*C. jacobsoni* Macfie, 1934	GN3.a, GN5.d, GN6.a, GN6.b	Potential vector of Bluetongue virus [61] and *Tibet Orbivirus* [62]; potential vector of *Leishmania* [75]; human biting [18]
*C. kusaiensis* Tokunaga, 1940	GN4.a, GN4.b, GN5.e	Human biting [18]
*C. obscurus* Tokunaga and Murachi, 1959	MA1, MA4, MA5, NA1	Human biting [18]
*C. okinawensis* Arnaud, 1956	GN1.a, GN1.b, GN2.a, GN4.a, GN4.b, GN5.a, GN5.b, GN5.d, GN5.e, GN5.f, GN5.g, GN5.h, GN5.d, GN6.a	–
*C. orientalis* Macfie, 1932	NA1	Potential vector of Bluetongue virus [77]; transmission of filarial nematode *Onchocerca gibsoni* [18]*;* potential vector of *Leishmania* [75]; human biting [18]
*C. oxystoma* Kieffer, 1910	GN1.a, GN1.c, GN3.b	Potential vector of Bluetongue virus [2]; potential vector of filarial nematode *Onchocerca gibsoni* [18]; vector of Epizootic haemorrhagic disease of deer [78]; potential vector of *Leishmania* [67, 75]; human biting [65]
*C. peliliouensis* Tokunaga, in Tokunaga & Esaki, 1936	GN3.a, GN5.g, GN5.e, GN5.f, GN5.h, GN5.i, GN5.j, MA1, MA3	Human biting [18]
*C. peregrinus* Kieffer, 1910	GN1.a, GN1.c, GN3.a, GN3.b, MA1, MA2. MA3	Potential vector of Bluetongue virus [2]; potential vector of filarial nematode *Onchocerca gibsoni* [79]; potential vector of *Leishmania* [67]; human biting [18, 65]
*C. perornatus* Delfinado, 1961	GN1.b, GN2.a, GN3.a, GN5.g, GN5.a, GN5.b, GN5.d, GN5.e, GN5.h, GN6.a	–
*C. quatei* Wirth & Hubert 1989	MA1, MA2, MA4, NA1	Human biting [18]
*C. shortti* Smith & Swaminath, 1932	GN1.b, GN5.d, GN5.e, MA2	Potential vector of filarial nematode *Onchocerca gibsoni* [18]; human biting [18]
*C. sumatrae* Macfie, 1934	GN1.c, GN2.a, GN5.a, GN5.b, GN5.f, GN5.g, GN5.h, GN6.a, GN6.b, MA6, NA1	Human biting [18]

***Culicoides*** **Subgenus** ***Trithecoides*** **Wirth and Hubert, 1959:2**

**Diagnostic characters.** Radial cells are well developed, particularly cell r_2_, which is included in a pale spot; scutum and upper pleuron of most species yellow and noticeably paler than lower pleuron; 3 well-developed sclerotized spermathecae present with a sclerotized ring at the duct junction.

***Culicoides barnetti*** **Wirth and Hubert, 1959:32**

**Diagnostic characters.** Female: Yellow mesonotum; SCo present on antennal segments 1, 9–13; prominently banded hind femur; hind leg with prominent blackish knee spot; pale haltere with prominently marked wing with broad pale apex.

**Distribution**: Indonesia, Malaysia, Papua New Guinea, Philippines, Singapore, Solomon Islands, Thailand, India (this study).

***Culicoides gouldi*** **Wirth and Hubert, 1989:139**

**Diagnostic characters.** Female: The only Asian species of subgenus *Trithecoides* having SCo distribution 1, 9, 11–13, lacking on segment 10 but present on 9.

**Distribution**: Indonesia, Malaysia, India (this study).

**Remarks**: Apex of hind femur was dark instead of pale and knee of foreleg darkened. However, all other characters examined such as AR, PR, number of mandibular teeth, P/H ratio, and spermathecae shape and size (Supplementary Table 1) are consistent with Wirth & Hubert, 1989 [18].

***Culicoides flaviscutellaris*** **Wirth and Hubert, 1989:132**

**Diagnostic characters.** Female: Pale mesonotum and scutellum; wing devoid of macrotichia, cell r_2_ broad and almost 2.5 times longer than r_1_ cell, wing base dark; mandibles with 12 sub-equal teeth; SCo present on antennal segments 1, 9–13; haltere pale; hind femur mostly pale with dark bands; spermathecae unequal, large one broader than long, conoidal, smaller ones subspherical.

**Distribution**: Malaysia, India (this study).

**Subgenus** ***Avaritia*** **Fox 1955:218**

**Diagnostic characters.** SCo usually present on segments 1, 9–13; poststigmatic pale spot usually covers the tip of cell r_2_ for short distance or almost half; distal pale spot usually filling apex of cell r_3_; veins M_1_, M_2_, M_3+4_ and Cu_1_ have dark apices at the wing margin.

***Culicoides flavipunctatus*** **Kitaoka 1975:199**

**Diagnostic characters.** Female: Adults relatively small in size; moderately distinct wing pattern, an isolated dark marking present sub-basally over vein M_2_ (Fig. 4, *C. flavipunctatus*) that is sometimes connected with a distal dark marking over vein M_2,_ (Fig. 4, *C. flavipunctatus* var.) stigmatic dark marking longer than basal dark marking on the costa.

**Distribution**: Indonesia, Laos, Timor-Leste, Malaysia, Japan, China, Papua New Guinea, Thailand, India (this study).

***Culicoides hui*** **Wirth & Hubert 1961:16**

**Diagnostic characters.** Female: Only two species of subgenus *Avaritia* have two pale areas distal to post stigmatic pale spot in cell r_3_: *C. hui* and *Culicoides annandalei* Majumdar and Das Gupta, *in* Gangopadhyay and Das Gupta 2000. However, *C. hui* differs from *C. annandalei* by having an infuscated haltere, wing length (0.83 mm-0.85 mm), AR (1.12–1.13), PR (2.4–2.6) and P/H (0.77).

**Distribution**: Indonesia, Laos, Timor-Leste, Malaysia, Thailand, Taiwan, China, Japan, Papua New Guinea, India (this study).

***Culicoides obscurus*** **Tokunaga and Murachi 1959:347**

**Diagnostic characters.** Female: Only species of subgenus *Avaritia* with a combination of following characters: poorly marked wings, presence of unbanded dark legs, dark brown haltere, SCo present on segments 1, 9–13, wing length (0.70–0.91 mm) and number of mandibular teeth (13–18).

**Distribution**: Indonesia, Papua New Guinea, Malaysia, Solomon Islands, Thailand, Australia, Timor-Leste, India (this study).

**Remark**: Wing pattern of our specimens was similar to obscurus (overt pattern) as described by Dyce et al. [55]. However, characters such as SCo distribution, AR, wing length, CR, PR, and P/H ratio was consistent to that of both Dyce et al. [55] and Wirth and Hubert [18].

**Subgenus** ***Meijerehelea*** **Wirth and Hubert, 1961:23**

**Diagnostic characters.** SCo usually present on segments 1–12, sometimes absent on some or all of the segments 5–8; cell r_2_ dark to apex, 2 pale spots on m_1_ cell, m_2_ cell without pale spot in front of mediocubital fork but with two pale spots lying distal to this fork, veins more or less narrowly pale marginated; 1 saclike or sagittate spermathecae present, sometimes with a very elongated duct.

***Culicoides histrio*** **Johannsen, 1946: 190**

**Diagnostic characters**. Female: The only Southeast Asian species having a crescent-shaped poststigmatic spot, extending posteriorly towards the apex of cell r_3_ and with SCo on antennal segments 1–12.

**Distribution**: Australia, Indonesia, Malaysia, Micronesia, Papua New Guinea, Philippines, Sri Lanka, Thailand, India (this study).

***Culicoides guttifer*** **(Meijere, 1907):209**

**Diagnostic characters**. Female: Pale spot posterior to r_2_ cell touching vein M_1_; single saclike spermatheca, with fine transverse wrinkled band on mid-portion and presence of minute hyaline punctures on distal 2/3 of spermatheca.

**Distribution**: Australia, Brunei, Indonesia, China, Laos, Malaysia, Philippines, Papua New Guinea, Thailand, Vietnam, India (this study).

### Subgenus unplaced

**Clavipalpis group Wirth & Hubert 1989:364**

**Diagnostic characters.** SCo present on proximal antennal segments but absent from segments 9–13; r_3_ cell with a small round pale spot at its apical or subapical region, variable in shape, usually diagnostic for species without any pale spot between it and distal poststigmatic spots, m_2_ cell always with pale spot lying in front of mediocubital fork, usually only one pale spot distad of fork; hind tibial comb usually with 4 spines.

***Culicoides perornatus*** **Delfinado, 1961:651**

**Diagnostic characters.** Female: The only species of Clavipalpis group with a more or less “U” shaped subapical pale spot present on the r_3_ cell and arm of “U” not meeting the wing margin; SCo present on antennal segments 1, 5–8, two per segment.

**Distribution**: Indonesia, Philippines, Papua New Guinea, Solomon Islands, India (this study).

**Ornatus group Wirth & Hubert** **1989:293**

**Diagnostic characters.** Penultimate segment of the antennal flagellomere bears 4–5 sensilla arranged in a distal ring; r_2_ cell moderately long, generally dark to the tip, though distally pale in some species; hind tibial comb usually with 4 spines (rarely 5) with one nearest to spur longest; 2 large sclerotized spermathecae present along with a vestigial one.

***Culicoides okinawensis*** **Arnaud, 1956:118**

**Diagnostic characters.** Female: Numerous macrotichia on wing, r_2_ cell totally included in dark stigmatic spot; two pale spots present on wing, one over r-m cross vein and the other distal to r_2_ cell (poststigmatic pale spot); SCo on antennal segments 1–12, double on most segments, multiple on 1 and 12.

**Distribution**: Indonesia, Japan, Laos, Taiwan, Thailand, Vietnam, India (this study).

***Culicoides quatei*** **Wirth & Hubert 1989:333**

**Diagnostic characters.** Female: The only species belonging to the Ornatus group having SCo on 1–12 with long prominent tufts; wing with apical pale spot on r_3_ cell not touching the wing margin; haltere pale;12–16 well developed mandibular teeth; 3rd palpal segment moderately swollen distally with small deep sensory pit.

**Distribution**: Indonesia, Malaysia, Thailand, India (this study).

**Coronalis group Dyce et al. 2007:41**

**Diagnostic characters.** Wings with a faint pattern, featuring a dark marking centered over the r-m crossvein; 2 functional spermathecae with trifurcated spermathecal ducts present.

***Culicoides coronalis*** **Lee and Reye, 1955:234**

**Diagnostic characters.** Female: SCo present on 1, 5–8, wing with obscure pale markings with a dark spot over r-m cross vein and presence of short, sparse, spine like macrotrichia on wings.

**Distribution**: Indonesia, Philippines, Australia, Papua New Guinea, India (this study).

Remarks: Hind tibial comb with five spines as observed in Indonesian samples reported by Wirth and Hubert, 1989 [18].

**Kusaiensis Group Dyce et al. 2007:44**

**Diagnostic characters.** Clavate 3rd palpal segment with a large, shallow sensory pit on distal half; hind tibial comb with 5–7 spines, where either second or first and second spines from spur longest; unpatterned wing.

***Culicoides kusaiensis*** **Tokunaga 1940:215**

**Diagnostic characters.** Female: Unpatterned wing with moderately numerous macrotichia on distal 2/3 portion, radial vein deeply infuscated; mandible with 10–12 teeth; SCo distributed on 1, 3, 5, 7, 9–12.

**Distribution**: Micronesia, China, Seychelles Island, Papua New Guinea, Indonesia, Malaysia, Thailand, India (this study).

## Discussion

The Andaman and Nicobar Islands are a remarkable repository of biodiversity with 27% of the terrestrial fauna in the archipelago being endemic [48]. Studies on invertebrates, particularly insects, have been inconsistent throughout the archipelago in comparison with avifauna or other megafauna. Nevertheless, 24% of the 2506 recorded insect species in this region appear to be endemic [48]. Considering haematophagous dipteran vectors in the Andaman and Nicobar (A&N), research has traditionally been focused on mosquitoes with two endemic species, namely, *Uranotaenia christophersi* Barraud and *Aedes seampi* Huang [57], and more recently, phlebotomine sand flies have also drawn attention [58]. However, studies on *Culicoides* midges, family Ceratopogonidae, have been neglected in the archipelago despite being a group of medico-veterinary and ecological significance. This study provides the first account of the *Culicoides* fauna in different habitats of the Andaman and Nicobar Islands (Tables 1, 2).

Recent records of the circulation of multiple BTV serotypes and the rising trend of livestock population in the A&N Islands highlighted the importance of clarification of the BTV vectors found in this island ecosystem [51, 53]. Information on the presence/absence of vector species and their composition is paramount in disease management and preventative practice. This preliminary survey reports 23 *Culicoides* species from the islands, 13 of which are recorded for the first time from India_._ Most of the specimens were trapped on light sheets (LS), in CDC traps, and as biting collections (BC) (Tables 1, 2). Only a negligible number of *Culicoides peliliouensis* Tokunaga, 1936 were trapped in MT (Tables 1, 2). The large proportion of new records for India may be explained by the relative lack of surveys in coastal, mangrove, and forested areas of mainland India and the relatively small distance of the A&N Islands from other Southeast Asian countries [59]. The newly recorded species are all present in other Southeast (SE) Asian countries.

Biting midges are important vectors and responsible for the transmission of various pathogens [3]; some of them have a significant impact on animal health [2]. Bluetongue is an economically important disease found throughout India in which seven putative vectors are thought to playing an important role in pathogen transmission [60]. *Culicoides actoni*, *C. peregrinus*, and *C. oxystoma* are regarded as potential vectors of BTV and other pathogens throughout the world [2], thus it is likely they will act in a similar role on these islands. *Culicoides jacobsoni* is widespread throughout Asia and is now regarded as a potential vector of BTV [61], as well as *Tibet Orbivirus* [62]. The threat it poses to livestock disease transmission in these islands requires assessment. Two bird-biting species, *C. histrio* and *C. guttifer*, belonging to the subgenus *Meijerehelea*, were also recorded in this survey, but their potential impact on various endemic birds in the islands is unknown. Additionally, we set up a CDC trap close to a breeding mound of Nicobar Megapode (scrubfowl) [Table 1, Site no: GN5f; Fig. 2E], an endangered ground nesting bird endemic to the Nicobar group of islands, and collected seven species of *Culicoides*, namely *C. barnetti*, *C. flavipunctatus*, *C. histrio*, *C. okinawensis, C. boophagus*, *C. peliliouensis*, and *C. sumatrae*. Blood-fed specimens will be analyzed in the future to determine whether any of these species feed on the megapode.

*Culicoides* have been recognized as serious human pests, causing widespread nuisance that can have serious repercussion on tourism, forestry, and agriculture [1]. The Andaman and Nicobar Islands are well known for their tourist attractions, which play a major role in the local economy. The Indian government [National Institute of Transforming India (NITI) Aayog] has initiated various measures for these islands to facilitate and boost tourism (https://aniidco.and.nic.in/announcement/PIM%20Andaman.pdf). During the study, *C. quatei*, *C. coronalis*, and *C. obscurus* from the Andaman Islands, and *C. barnetti* from the Great Nicobar Island, were witnessed to bite the authors/volunteers. Man-biting habits of *C. quatei*, *C. obscurus*, *C. coronalis*, and *C. barnetti* were also reported by Wirth and Hubert (1989) [18] and Bellis (2013) [63]. Moreover, *C. obscurus* was recently found to include several cryptic species and warrants further investigation to determine the status of this species and its pest potential [64]. Human biting species such as *C. shortti*, *C. jacobsoni*, *C. sumatrae*, *C. peliliouensis*, *C. huffi*, *C. oxystoma*, *C. peregrinus*, *C. gouldi*, and *C. kusaiensis* were found in the survey area [18, 63, 65], although of these, only *C. peliliouensis* has been reported as being a significant human pest [66]. Nonetheless, there is a need to explore the role of these species and their potential nuisance effect on local populations and tourism. Additionally, evidence of anthropophagic behavior [65] and the putative role of *C. oxystoma*, *C. peregrinus*, and *C. huffi* as potential vectors of *Leishmania* in Thailand [67], highlights a possible threat of Leishmanial transmission on local human population and tourists.

Our surveys included different habitats viz., coastal, mangrove, and forested areas of the A&N Islands (Tables 1 and 2). Finding of high proportion of new records in this study may be explained due to limited/absence of study in these habitat types. India is a mega diversity country with heterogeneous habitats, however, *Culicoides* diversity is relatively low compared with much smaller countries such as Thailand [19], thus it can be assumed that exploration of different habitats in mainland India will increase the species checklist. Among the newly recorded species, *C. coronalis*, *C. kusaiensis*, *C. flavipunctatus*, *C. histrio*, *C. quatei*, *C. obscurus*, and *C. perornatus* are apparently associated with seashores or beaches [18, 19, 63], and our observations regarding these maritime species (Tables 1, 2) aligned with previous observations. Limited information exists regarding the distribution and biology of *C. histrio*, *C. hui*, and *C. okinawensis*, and these species are apparently associated with mangroves [18, 19, 63]. We also observed the association of these species with mangrove forests in our study (Tables 1, 2). A total of four *Culicoides* species, namely *C. hui*, *C. flavipunctatus*, *C. quatei*, and *C. kusaiensis*, were also recently newly recorded from Thailand [19]. The similarity of species composition, again, may be due similarity of study sites, as both studies included beaches and mangrove forests. *Culicoides flaviscutellaris*, a species belonging to subgenus *Trithecoides,* was trapped from areas with human settlements in our study (Tables 1, 2). This is only the second report of this species since its initial documentation from Malaysia [18]. Among the species reported in this study, most were collected from areas in and around human settlements (16 spp.) followed by mangrove (15 spp.), seashore/beach (15 spp.), and forested areas (13 spp.), with *C. barnetti*, *C. perornatus*, *C. okinawensis*, and *C. huffi* being common in all the sites (Tables 1, 2). However, keeping in mind of flight ranges of *Culicoides* species [68], differential attraction to light traps [69], and diverse habitat types within short distances in the A&N Islands (Table 1), further study is required to strengthen the observations regarding associations of species with particular habitats.

A regular update of economically important groups of insects is pivotal for informing risk-management decision relating to health and nuisance pests, maintaining expertise to overcome the bottleneck of taxonomic challenges, assisting with conservation effort, and assessing the vulnerability to the introduction and colonization of exotic species [70]. The updated checklist includes 93 species of *Culicoides* in India (Table 3), among them 13 recorded for the first time from the country. All 23 species found in this island ecosystem are reported from neighboring countries in SE Asia, thus their presence is not surprising. After careful revision, it was found that some of the species, i.e., *Culicoides bolitinos* Meiswinkel, *Culicoides azerbajdzhanicus* Dzhafarov, *Culicoides baueri* Hoffman, and *Culicoides punctatus* (Meigen), which were mentioned earlier in checklists from India [45], are not included in this checklist, as the presence of those species was not supported by any published or grey literature. Chatterjee et al.[46] proposed *Culicoides himalayae* Kieffer, *Culicoides molestior* Kieffer, *Culicoides odiosus* Kieffer, and *C. magnificus* Sen & Gupta as nomen dubium; nevertheless, this requires further study, thus we have retained these species on our checklist. Furthermore, *Culicoides aequalispinus* Nandi, Mazumdar, & Das Gupta, *Culicoides fuscitibialis* Nandi, Mazumdar, & Das Gupta, and *Culicoides pateli* Nandi, Mazumdar, & Das Gupta in the Ornatus group have somehow been overlooked by authors of previous checklists [45, 46]. *Culicoides schultzei*, an Afrotropical species; has been previously reported from India [71, 72], however, it is widely accepted that this species does not occur in Asia [60, 73], thus we have not included this species in the checklist. *Culicoides sumatrae* is recorded from Kerala, in the southwest of mainland India [37], and the A&N Islands, thus it would be surprising if this species is not more widespread in the country.

**Table 3:** Updated checklist of Indian *Culicoides* fauna

Species	Type Locality	Distribution (states or union territories) in India	Reference of original description	References of records from India
Subgenus ***Avaritia*** Fox,1955				
* C. actoni* Smith, 1929*	India (Assam: Burnihat)	AP, AS, BR, KA, KL, MH, ML, TN, TS, WB	[27]	[6, 27, 37, 60, 80, 81]
* C. annandalei* Majumdar & Das Gupta, in Gangopadhyay & Das Gupta, 2000	India (Sikkim: Renock)	SK	[37]	[37]
* C. autumnalis* Sen & Das Gupta, 1959	India (West Bengal: Dum Dum)	WB	[6]	[6, 37]
* C. boophagus* Macfie, 1937	Malaysia (Kuala Lumpur)	KA, KL	[105]	[37, 80]
* C. brevitarsis* Kieffer, 1917*	Australia	TN, WB	[82]	[33, 37, 44, 60, 82]
* C. certus* Das Gupta, 1962	India (West Bengal: Calcutta)	WB	[32]	[32]
* C. definitus* Sen & Das Gupta, 1959	India (West Bengal: Dum Dum)	WB	[6]	[6]
* C. dumdumi* Sen & Das Gupta, 1959	India (West Bengal: Dum Dum)	WB	[6]	[6, 37]
***C. flavipunctatus*** **Kitaoka, 1975**	Japan (Yonaguni Island: Sonai)	AN	[106]	This study
*C. fulvus* Sen & Das Gupta, 1959*	India (West Bengal: Dum Dum)	KA, TN, WB	[6]	[6, 65, 80, 83]
***C. hui*** **Wirth & Hubert, 1961**	Taiwan (Pingtung: Utai)	AN	[107]	This study
*C. imicola* Kieffer, 1913*	East Africa (Kenya: Mombasa Tiwi)	JH, KA, KL, RJ, TN, TS, WB	[108]	[37, 60, 84–86]
*C. inexploratus* Sen & Das Gupta, 1959	India (West Bengal: Dum Dum)	WB	[6]	[6, 37]
*C. jacobsoni* Macfie, 1934	Indonesia (Sumatra: Bukittinggi)	KL	[109]	[37]
* C. orientalis* Macfie, 1932*	Indonesia (East Java)	KA, SK, TN, WB	[30]	[6, 30, 37, 80]
* C. sikkimensis* Das Gupta, 1963	India (Sikkim)	SK	[34]	[34]
* C. himalayae* Kieffer, 1911	India (West Bengal: Kurseong)	SK, WB	[22]	[6, 22]
* C. molestior* Kieffer, 1911	India (West Bengal: Calcutta)	WB	[23]	[6, 21, 23]
* C. odiosus* Kieffer, 1910	India (West Bengal: Calcutta)	WB	[21]	[6, 21]
***C. obscurus*** **Tokunaga and Murachi, 1959**	Indonesia (Sumatra)	AN	[7]	This study
Subgenus ***Beltranmyia*** Vargas 1953				
* C. circumscriptus* Kieffer, 1918	Tunisia (Tunis)	BR, KA, OR, WB	[110]	[37, 84, 87]
***Chaetopthalmus*** group Amosova 1957				
* C. majorinus* Chu, 1977	China (Tibet)	AP, WB	[111]	[39, 88, 89]
* C. yadongensis* Chu, 1984	China (Tibet)	WB	[89]	[39, 88]
* C. ateripes* Nandi & Mazumdar, 2014	India (West Bengal: Darjeeling)	WB	[39]	[39]
***Clavipalpis*** group Wirth & Hubert 1989				
* C. clavipalpis* Mukerji, 1931	India (West Bengal: Calcutta)	BR, KA, KL, TN, WB	[28]	[6, 18, 28, 37, 41, 44, 72, 80]
* C. distinctus* Sen & Das Gupta, 1959	India (West Bengal: Dum Dum)	KL, WB	[6]	[6, 37, 41]
* C. mukerjii* Majumdar and Das Gupta, *in* Gangopadhyay and Das Gupta, 2000	India (West Bengal: Habra)	WB	[37]	[37, 41]
* C. soleamaculatus* Nandi & Mazumdar, 2014	India (West Bengal: Habra)	WB	[41]	[41]
* C. similis* Carter, Ingram & Macfie, 1920	Ghana (Gold Coast: Accra and Oblogo)	JH, KA, TN, WB	[112]	[41, 60, 80]
***C. perornatus*** **Delfinado, 1961**	Philippines (Mindanao)	AN	[8]	This study
* C. pseudosimilis* Saha et al. 2017	India (West Bengal)	WB	[90]	[90]
* C. causeyi* Majumdar & Das Gupta, *in* Gangopadhyay & Das Gupta, 2000	India (West Bengal)	WB	[37]	[37, 41]
* C. huffi* Causey, 1938	Thailand (Bangkok)	KA, KL, OR, TN, WB	[5]	[37, 41, 60, 65, 84, 91]
***Coronalis*** group Dyce et al. 2007				
*C. coronalis* Lee & Reye, 1955	Australia (Queensland: Prince of Wales Island)	AN	[113]	This study
Subgenus ***Haemophoructus*** Macfie 1925				
*** C. rariradialis*** **Das Gupta, 1963**	India (Sikkim: Nayabazar)	SK	[34]	[34]
Subgenus ***Hoffmania*** Fox 1948				
* C. indianus* Macfie, 1932	India (Karnataka: Dharwar)	KA, MH	[30]	[6, 30, 37, 92]
* C. isoregalis* Majumdar and Das Gupta, *in* Majumdar et al*.* 1997	India (West Bengal: Darjeeling)	WB	[36]	[36, 92]
* C.neoregalis* Majumdar and Das Gupta, *in* Majumdar et al*.* 1997	India (West Bengal: Darjeeling)	WB	[36]	[36, 92]
* C. paraliui* Das Gupta, 1962	India (West Bengal: Dum Dum)	WB	[32]	[32]
* C. recurvus* Delfinado, 1961	Philippines (Luzon: Pampanga)	AS	[8]	[18, 37]
* C. sumatrae* Macfie, 1934	Indonesia (Sumatra: Bukittinggi)	KL	[114]	[37]
* C. pseudoregalis* Majumdar and Das Gupta, *in* Majumdar et al*.* 1997	India (West Bengal: Darjeeling)	WB	[36]	[36, 92]
* C. quasiregalis* Majumdar and Das Gupta, *in* Majumdar et al*.* 1997	India (West Bengal: Darjeeling)	WB	[36]	[36, 92]
* C. regalis* Majumdar and Das Gupta, *in* Majumdar et al*.* 1997	India (West Bengal: Darjeeling)	WB	[36]	[36, 92]
* C. proximus* Nandi & Mazumdar, 2013	India (West Bengal: Habra)	WB	[92]	[92]
* C. subregalis* Majumdar and Das Gupta, *in* Majumdar et al*.* 1997	India (West Bengal: Darjeeling)	WB	[36]	[36, 92]
* C. peregrinus* Kieffer, 1910*	India (Orissa: Puri)	AP, AS, KA, KL, OR, JH, MH, TN, TS, WB	[21]	[21, 37, 65, 72, 84, 85]
* C. innoxius* Sen & Das Gupta, 1959	India (West Bengal: Calcutta)	AP, BR, JH, KA, KL, TN, WB	[6]	[6, 37, 60, 65, 84, 85, 89]
* C. pararegalis* Majumdar & Das Gupta *in* Majumdar et al*.* 1997	India (West Bengal: Darjeeling)	WB	[36]	[36, 92]
***Kusaiensis*** Group Dyce et al.2007				
***C. kusaiensis*** **Tokunaga, 1940**	Caroline Islands	AN	[115]	This study
Subgenus ***Meijerehelea*** Wirth & Hubert 1961				
* C. arakawae* (Arakawa, 1910)	Japan (Honshu: Aichi)	AS, KA, KL, MH, WB	[116]	[18, 37, 84]
***C. guttifer*** **(de Meijere, 1907)**	Indonesia (Java: Semarang)	AN	[97]	This study
* C. hegneri* Causey, 1938	Thailand (Bangkok)	WB	[5]	[37, 65]
***C. histrio*** **Johannsen, 1946**	Guam (Piti)	AN	[38]	This study
* C. magnithecalis* Majumdar & Das Gupta, *in* Gangopadhyay & Das Gupta, 2000	India (West Bengal: Siliguri)	WB	[37]	[37]
Subgenus ***Monoculicoides*** Khalaf 1954				
* C. rarus* Das Gupta, 1963	India (Sikkim: Nayabazar)	SK	[34]	[34, 37]
* C. homotomus* Kieffer, 1922	Taiwan (Daitotei)	KA, WB	[117]	[37, 93]
* C. obtusus* Chatterjee, Brahma & Hazra, 2020	India (West Bengal: Birbhum)	WB	[46]	[46]
Subgenus ***Oecacta*** Poey 1853				
* C. distinctipalpis* Majumdar & Das Gupta, *in* Gangopadhyay & Das Gupta, 2000	India (West Bengal: Raniganj)	WB	[37]	[37]
***Ornatus*** group Wirth & Hubert 1989				
* C. aequalispinus* Nandi, Mazumdar & Das Gupta, 2014	India (West Bengal: Siliguri)	WB	[94]	[94]
* C. fuscitibialis* Nandi, Mazumdar & Das Gupta, 2014	India (West Bengal: Darjeeling)	WB	[94]	[94]
***C. okinawensis*** **Arnaud, 1956**	Japan (Okinawa: Sukiran Area)	AN	[118]	This study
* C. pateli* Nandi, Mazumdar & Das Gupta, 2014	India (West Bengal: Gobardanga)	WB	94]	[94]
* C. peliliouensis* Tokunaga, in Tokunaga & Esaki, 1936	Palau (Peliliou Island)	TN, WB	[95]	[60, 95, 96]
***C. quatei*** **Wirth & Hubert, 1989**	Malaysia (Selangor: Rantaupanjanj)	AN	[97]	This study
Subgenus ***Pontoculicoides*** Remm 1968				
* C. kamrupi* Sen & Das Gupta, 1959	India (Assam: Gauhati)	AS	[6]	[6]
Subgenus ***Remmia*** Glukhova 1977				
* C. oxystoma* Kieffer, 1910*	India (West Bengal: Calcutta)	AP, AS, KA, DL, KL, BR, TN, TS, JH, WB, OR, MH, HP,HR,PB, RJ, GJ	[21]	[21, 37, 60, 84, 85, 97–100]
***Shermani*** group Wirth & Hubert 1989				
* C. dryadeus* Wirth and Hubert, 1972	Malaysia (Selangor: Ampang Forest Reserve)	WB	[101]	[90, 101]
* C. kepongensis* Lee, 1988	Taiwan	TN	[102]	[60]
* C. selangorensis* Wirth and Hubert, 1989	Malaysia (Selangor: Sungai Buloh Forest Researve)	WB	[97]	[102]
* C. cornus* Chatterjee, Brahma & Hazra, 2020	India (West Bengal: Purba Bardhaman)	WB	[46]	[46]
***Shortti*** group Wirth & Hubert 1989				
* C. swaminathi* Majumdar & Das Gupta, *in* Gangopadhyay& Das Gupta, 2000	India (West Bengal: Habra)	WB	[37]	[37, 40]
* C. shortti* Smith & Swaminath, 1932	India (Assam: Gauhati)	AS, WB	[29]	[29, 40]
* C. rectilis* Nandi & Mazumdar, 2014	India (West Bengal: Habra)	WB	[40]	[40]
Subgenus ***Trithecoides*** Wirth & Hubert 1959				
***C. barnetti*** **Wirth & Hubert, 1959**	Malaysia (Ulu Langat: Selangor)	AN	[104]	This study
***C. gouldi*** **Wirth & Hubert, 1989**	Malaysia	AN	[97]	This study
* C. inciderus* Nandi, Mazumdar & Chaudhuri, 2015	India (West Bengal: Habra)	WB	[43]	[43]
* C. parararipalpis* Das Gupta,1963	India (Sikkim: Nayabazar)	SK	[34]	[34, 37, 43]
* C. anopheles* Edwards, 1922	Malaysia (Pudoh Gaol: Kuala Lumpur)	AP, AS, BR, JH, KA, KL,MP, PB, TN, WB, TL	[26]	[26, 37, 43, 85, 103]
* C. inornatithorax* Das Gupta, 1963	India (Sikkim: Nayabazar)	SK, ML	[34]	[34, 43, 81]
***C. flaviscutellaris*** **Wirth & Hubert, 1989**	Malaysia	AN	[97]	This study
* C. flaviscutatus* Wirth & Hubert, 1959	Indonesia (Borneo: Labuan Island)	AS, MH, KL, WB	[104]	[37, 97, 104]
* C. raripalpis* Smith, 1929	India (Assam: Burnihat)	AS, KL, SK, WB	[27]	[27, 34, 37, 43, 97]
* C. insolens* Chaudhuri & Das Gupta, 1986	India (West Bengal: Darjeeling)	WB	[35]	[35, 43]
* C. forcepifinis* Nandi, Mazumdar & Chaudhuri, 2015	India (West Bengal: Habra)	WB	[43]	[43]
* C. macfiei* Causey, 1938	Thailand (Chiengrai)	KL, WB	[5]	[6, 34, 97]
* C. palpifer* Das Gupta & Ghosh, 1956	India (West Bengal: Thakurpukur)	AS, KA, WB	[31]	[6, 31, 37, 84, 97]
* C. tympanus* Nandi, Mazumdar & Chaudhuri, 2015	India (West Bengal: Jhargram)	WB	[43]	[43]
***Williwilli*** group Wirth & Hubert, 1989				
* C. palpisimilis* Wirth & Hubert, 1989	Malaysia	KA	[97]	[80, 94]
Miscellaneous unplaced species				
* C. turgidus* Sen & Das Gupta, 1959	India (West Bengal: Calcutta)	WB	[6]	[6]
* C. peculiaris* Majumdar & Das Gupta, *in* Gangopadhyay & Das Gupta, 2000	India (West Bengal: Darjeeling)	WB	[37]	[37]
* C. mesghalii* Navai, 1973	Iran (Persian Gulf: Zyarat-Ali)	TN	[119]	[60]
* C. magnificus* Sen & Gupta, 1959	India (West Bengal: Calcutta)	WB	[6]	[6]

Abbreviations of Indian states/union territories: Andaman and Nicobar-AN, Andhra Pradesh-AP, Assam-AS, Bihar-BR, Delhi-DL, Gujarat-GJ, Haryana-HR, Himachal Pradesh-HP, Jharkhand-JH, Karnataka-KA, Kerala-KL, Madhya Pradesh-MP, Maharashtra-MH, Meghalaya-ML, Orissa-OR, Punjab-PB, Rajasthan-RJ, Sikkim-SK, Tamil Nadu-TN, Telangana-TS, West Bengal- WB. ‘*’ denotes actual or potential bluetongue vectors from India. Species name in bold denotes new record to India

Although the survey encompassed a large area and multiple habitats of the Andaman and Nicobar Islands, the study was only confined to 2 month-long surveys during the same season i.e. September of 2022 and 2023. Trapping per site was also limited to 1–2 days, which could underestimate species diversity, particularly of species with seasonal population fluctuations. Besides, our collection methods were mainly based on LS and CDC data which may underestimate the diurnal species composition [74]. To overcome the shortfall of the study, more in-depth surveys should be conducted over different seasons and times of the year using various trapping procedures.

## Conclusions

The presence of *Culicoides* species in the A&N Islands including several potential vectors of BTV and the circulation of multiple BTV serotypes highlights the necessity for regular surveillance of the BTV vectors closely associated with livestock and to implement appropriate disease control measures where necessary. Furthermore, identifying potential nuisance pests is of utmost importance considering the significance of the islands as a tourism destination in India. The checklist of 93 valid *Culicoides* species, with their state distribution, provides a baseline for the development of a genetic library and the preparation of an atlas of diagnostic characters. Additionally, large proportion of the new records in this study emphasize the need for additional thorough surveys using various trapping techniques in different habitats across different seasons, which may further increase the number of *Culicoides* species from India.

## Supplementary Information

Supplementary material 1.

## Data Availability

No datasets were generated or analyzed during the current study.

## Abbreviations

BTV: Bluetongue virus
A&N: Andaman and Nicobar
MA: Middle Andaman
NA: North Andaman
GN: Great Nicobar
LS: Light sheet
MT: Malaise trap
BC: Biting collection
P/H: Proboscis/head ratio
CR: Costal ratio
AR: Antennal ratio
PR: Palpal ratio
SCo: Sensilla coeloconica
r_2_: 2nd radial cell
r_3_: 3rd radial cell
M_1_: 1st median vein
M_2_: 2nd median vein
Cu_1_: 1st cubital vein
r-m: Radial-medial crossvein
