# Supplementary material for: A preliminary account of Culicoides (Diptera: Ceratopogonidae) from the Andaman and Nicobar Islands with 13 new records and updated species inventory from India
Source: Parasit Vectors. 2025 Jan 17;18:15. doi: 10.1186/s13071-024-06614-w (PMC11740402; doi:10.1186/s13071-024-06614-w)
Supplement: Supplementary file 1 — Supplementary material 1. [file 13071_2024_6614_MOESM1_ESM.docx]

Supplementary Table 1: Material examined and morphometric measurements of *Culicoides* species found during this study.

| Species | Site Number | Material examined (♀) | Wing Length (mm) | Coastal Ratio (CR) | Palpal Ratio (PR) | Proboscis/head ratio (P/H) | Antennal Ratio (AR) | No. of mandibular teeth | Antennal Sensilla pattern | No. of hind tibial spine (spine nearest the spur longest) | No. of functional Spermatheca and size (length*breadth in mm) | Remarks |
| --- | --- | --- | --- | --- | --- | --- | --- | --- | --- | --- | --- | --- |
| *C. actoni*  (n=6) | GN1.b  GN1.c GN5.a  GN5.b  GN5.e | 1  1  1  1  2 | 0.71  (0.67-0.79) | 0.56  (0.53-0.59) | 2.23  (2.16-2.31) | 0.81  (0.76-0.84) | 1.20  (1.16-1.24) | 13-15 | 1, 10-13 | 5 (1^st^) | 2  0.0325*0.03  0.0325*0.027 | - |
| *C. barnetti* (n=11) | GN1.c  GN2.a GN2.b  GN3.b GN5.a GN5.b GN5.e GN5.f  GN5.g GN5.h GN6.a | 1  1  1  1  1  1  1  1  1  1  1 | 0.85  (0.83-0.87) (n=7) | 0.72  0.69-0.72)  (n=7) | 2.38  (2-2.66) | 0.59  (0.56-0.64) | 0.91  (0.88-0.95) (n=9) | 11-13 | 1, 9-13 | 4 (2^nd^) | 3  0.375*0.025  0.02*0.02  0.02*0.02 | - |
| *C. boophagus* (n=3) | GN5.a, GN5.b | 2  1 | 0.82  (0.79-0.84) | 0.60  (0.59-0.62) | 2.36  (2.14-2.66) | 0.69  (0.65-0.73) | 1.12  (1.09-1.18) | 11-12 | 1,9-13 | 5 (1^st^) | 2  0.055*0.04  0.037*0.032 | - |
| *C. coronalis* (n=2) | MA1^*^ | 2 | 0.91  (0.90-0.92) | 0.65  (0.63-0.67) | 2.1  (2.1-2.2) | 0.66  (0.64-0.68) | 1.29  (1.28-1.30) | 12-13 | 1, 5-8 | 5 (1^st^) | 2  0.055*0.375  0.05*0.325 | eyes with interfacetal hair |
| *C. flavipunctatus* (n=7) | GN5.f  GN5.a GN5.b  GN5.h | 2  3  1  1 | 0.77  (0.76-0.79) | 0.59  (0.57-0.62) | 2.12  (2.06-2.21) | 0.79  (0.78-0.81) | 1.38  (1.32-1.45) | 14-15 | 1, 9-13 | 5 (1^st^) | 2  0.04*0.037  0.04*0.03 | - |
| *C.* *flaviscutellaris* (n=3) | GN1.c, GN5.a, GN5.b | 1  1  1 | 0.85  (0.85-0.86) | 0.77  (0.76-0.78) | 2.59  (2.5-2.66) | 0.57  (0.55-0.59) | 0.80  (0.79-0.82) | 11-12 | 1, 9-13 | 4 (2^nd^) | 3  0.275*0.275  0.0125*0.015  0.015*0.015 | - |
| *C. gouldi*  (n=5) | GN2.b GN5.a GN5.b  GN5.e  GN5.h | 1  1  1  1  1 | 0.81  (0.79-0.83) | 0.71  (0.70-0.72) | 1.99  (1.87-2.14) | 0.53  (0.51-0.56) | 0.99  (0.97-1.01) | 12-13 | 1, 9, 11-13 | 4 (2^nd^) | 3  0.035*0.275  0.025*0.0175  0.02*0.0175 | - |
| *C. guttifer* (n=2) | GN5.b  NA1 | 1  1 | 1.02  (1-1.05) | 0.63  (0.61-0.66) | 2.44  (2.35-2.54) | 0.87 | 1.51  (1.5-1.52) | 13-14 | 1-4, (5), (6), 9-12 | 4 (1^st^) | 1  0.117*0.05 | - |
| *C. histrio* (n=5) | GN3.a  GN5.a GN5.b  GN5.e  GN5.h | 1  1  1  1  1 | 0.93  (0.92-0.95) | 0.60  (0.59-0.61) | 2.16  (2.07-2.27) | 0.76  (0.72-0.81) | 1.25  (1.21-1.29) | 12-13 | 1-12 | 4 (1^st^) | 1  0.062*0.04 | - |
| *C. huffi* (n=6) | GN3.b  NA1  GN6.a | 2  2  2 | 0.78  (0.76-0.81) | 0.54  (0.53-0.55) | 1.81  (1.77-1.87) | 0.56  (0.54-0.58) | 1.54  (1.49-1.61) | 9-10 | 1,3,5-8 | 4 (1^st^) | 2  0.06*0.035  0.05*0.03 | - |
| *C. hui* (n=2) | GN5.a GN5.b | 1  1 | 0.83  (0.82-0.84) | 0.61  (0.60-0.62) | 2.41  (2.33-2.5) | 0.78 | 1.09  (1.06-1.12) | 12-13 | 1, 9-13 | 5 (1^st^) | 2  0.05*0.0375  0.0375*0.0325 | - |
| *C. jacobsoni* (n=3) | GN5.d  GN6.a  GN6.b | 1  1  1 | 0.89  (0.87-0.91) | 0.64  (0.63-0.65) | 2.79  (2.71-2.85) | 0.8  (0.79-0.81) | 1.27  (1.25-1.29) | 16-17 | 1,9-13 | 5 (1^st^  ) | 2  0.045*0.03  0.005*0.03 | - |
| *C. kusaiensis* (n=5) | GN4.a  GN4.b GN5.e | 2  2  1 | 0.80  (0.79-0.81) | 0.61  (0.59-0.63) | 2.23  (2.17-2.27) | 0.91  (0.90-0.93) | 1.45  (1.43-1.47) | 11-12 | 1,3,5,7,9-12 | 5 (1^st^) | 2  0.09*0.04  0.105*0.04 | - |
| *C. obscurus* (n=4) | MA1*  MA4*  MA5  NA1 | 1  1  1  1 | 0.73  (0.72-0.75) | 0.64  (0.63-0.65) | 2.06  (2-2.12) | 0.79  (0.77-0.81) | 1.34 | 13-14 | 1, 9-13 | 5 (1^st^) | 2  0.0425*0.03  0.0425*0.03 | - |
| *C. okinawensis* (n=8) | GN4.a GN4.b  GN5.f GN5.g  GN6.a | 1  1  1  2  2 | 0.96  (0.93-1.02) | 0.62  (0.60-0.63) | 2.53  (2.33-2.68) | 0.89  (0.86-0.93) | 1.38  (1.33-1.43) | 16-18 | 1-12 (double Sco on 2, 4, 8, 11 and multiple on 1 and 12) | 4 (2^nd^) | 2  0.06*0.0475  0.06*0.05 | - |
| *C. orientalis* (n=2) | NA1 | 2 | 0.83  (0.83-0.84) | 0.61  (0.61-0.62) | 2.87  (2.85-2.89) | 0.95  (0.94-0.97) | 1.19  (1.19-1.20) | 17 | 1, 9-13 | 5 (1^st^) | 2  0.04*0.32  0.045*0.35 | - |
| *C. oxystoma* (n=3) | GN1.a  GN1.c GN3.b | 1  1  1 | 0.98  (0.96-0.99) | 0.52  (0.50-0.53) | 2.06  (2-2.08) | 0.64  (0.62-0.65) | 1.07  (1.01-1.06) | 12-13 | 1, 6-8 | 4 (1^st^) | 2  0.047*0.032  0.047*0.032 | - |
| *C. peliliouensis* (n=12) | GN3.a GN5.g GN5.e GN5.f GN5.h GN5.i GN5.j  MA1  MA3 | 2  1  1  1  1  1  1  2  2 | 1.04  (0.82-1.05) | 0.61  (0.52-0.65) | 2.04  (2-2.6) | 0.64  (0.61-0.68) (n=9) | 1.12  (1.02-1.15) (n=9) | 11-14 | 1-12, varied considerably often absence on 2, 3, 4, 8 | 4-5 (1^st^) | 2  0.0525*0.04  0.0525*0.0375 | For most of the specimens, sensillar presence/absence varies even on two antennal segments of single specimen |
| *C. peregrinus* (n=4) | GN3.a  GN3.b  MA1  MA2 | 1  1  1  1 | 1.07  (1.04-1.13) | 0.61  (0.60-0.63) | 2.9  (2.8-3.06) | 0.76  (0.73-0.78) | 1.14  (1.07-1.17) | 15-16 | 1, 9-13 | 6 (2^nd^) | 2  0.05*0.0375  0.05*0.0375 | - |
| *C. perornatus* (n=6) | GN1.b GN2.a  GN3.a  GN5.b  GN5.h  GN6.a | 1  1  1  1  1  1 | 0.94 (0.93-0.96) | 0.61  (0.60-0.63) | 1.93  (1.76-2.09) | 0.50  (0.48-0.52) | 1.62 (1.61-1.65) | 13 | 1, 5-8 (2 per segment) | 4 (1^st^) | 2  0.06*0.0375  0.045*0.03 | - |
| *C. quatei* (n=4) | MA1*  MA2  MA4*  NA1 | 1  1  1  1 | 0.83  (0.82-0.86) | 0.61  (0.60-0.62) | 2.11 (2.09-2.13) | 0.69  (0.67-0.71) | 1.18  (1.16-1.19) | 14-15 | 1-12 (tuft long and prominent) | 4 (1^st^) | 2  0.055*0.325  0.04*0.0375 | - |
| *C. shortti*  (n=4) | GN1.b  GN5.d GN5.e  MA2 | 1  1  1  1 | 0.88  (0.87-0.9) | 0.55  (0.54-0.56) | 2.63  (2.59-2.66) | 0.65  (0.65-0.66) | 0.97  (0.96-0.98) | 14-15 | 1, 6-8 | 5-6 (2^nd^) | 2  0.05*0.03  0.042*0.03 | - |
| *C. sumatrae* (n=11) | GN1.c  GN2.a GN5.a GN5.b GN5.f GN5.g GN5.h GN6.a GN6.b MA6  NA1 | 1  1  1  1  1  1  1  1  1  1  1 | 1.03  (0.99-1.12) | 0.69  (0.68-0.71) | 3.33  (3.21-3.5) | 0.81  (0.79-0.84) (n=7) | 1.06  (1.01-1.13) | 21-22 | 1, 9-13 | 5 (2^nd^) | 2  0.06*0.0425  0.045*0.0375 | 3^rd^ palpal segment with pit located distally |

Site Number refers to Table 1. ‘*’ denotes specimen examined from biting collection (BC).
